# Novel Wireless Sensor System for Dynamic Characterization of Borehole Heat Exchangers

**DOI:** 10.3390/s110707082

**Published:** 2011-07-08

**Authors:** Julio Martos, Álvaro Montero, José Torres, Jesús Soret, Guillermo Martínez, Raimundo García-Olcina

**Affiliations:** 1 Department of Electronic Engineering, Universidad de Valencia, Campus de Burjassot, CP 46100 Valencia, Spain; 2 Instituto de Ingeniería Energética, Universidad Politécnica de Valencia, Campus de Vera, CP 46022 Valencia, Spain; E-Mail: almonter@upvnet.upv.es; 3 Department of Electronic Engineering, Universidad de Valencia, Campus de Burjassot, CP 46100 Valencia, Spain; E-Mails: jose.torres@uv.es (J.T.); jesus.soret@uv.es (J.S.); guillermo.martinez@uv.es (G.M.); raimundo.garcia@uv.es (R.G.-O.)

**Keywords:** heat pumps, geothermal energy, thermal analysis, wireless sensors

## Abstract

The design and field test of a novel sensor system based in autonomous wireless sensors to measure the temperature of the heat transfer fluid along a borehole heat exchanger (BHE) is presented. The system, by means of two specials valves, inserts and extracts miniaturized wireless sensors inside the pipes of the borehole, which are carried by the thermal fluid. Each sensor is embedded in a small sphere of just 25 mm diameter and 8 gr weight, containing a transceiver, a microcontroller, a temperature sensor and a power supply. A wireless data processing unit transmits to the sensors the acquisition configuration before the measurements, and also downloads the temperature data measured by the sensor along its way through the BHE U-tube. This sensor system is intended to improve the conventional thermal response test (TRT) and it allows the collection of information about the thermal characteristics of the geological structure of subsurface and its influence in borehole thermal behaviour, which in turn, facilitates the implementation of TRTs in a more cost-effective and reliable way.

## 1. Introduction

In recent years the growth in the number of air conditioning systems driven by ground source heat pumps (GSHP) or geothermal heat pumps (GHP) is estimated between 10% and 30% each year [1]. The air conditioning systems based on ground heat exchange can provide both heating and cooling while reducing the electrical consumption and increasing the efficiency of the system [2]. Commercial vendors claim that geothermal systems in residential applications can save homeowners 40 to 70% in heating costs and 30 to 50% in cooling costs compared to conventional systems [3], and those claims seems to be in good agreement with the results shown in the literature. Urchueguia *et al.* [2] showed a heating cost reduction of 43% and a cooling cost reduction of 37% in a real experiment carried out over a whole year.

A geothermal heat pump is a heat pump which uses the subsurface as source or sink of heat, being a more convenient energy source. The most remarkable effect of the use of ground heat exchange through a borehole heat exchanger (BHE) is the reduction of electrical energy consumption, compared with the consumption of the standard technology based on air heat exchange. Therefore, CO_2_ emissions associated with the use of electric power decreases substantially [4,5]. The European Community and other international agencies, as the U.S. Department of Energy or the American International Energy Agency, are considering GSHP in the field of “heat production from renewable sources”.

Additionally, it has been demonstrated that energy efficiency potential improvement in air conditioning systems based on GSHP can be as large as 30%, by performing a proper management of the different equipment making up the air conditioning system [6]. Moreover, this energy efficiency potential improvement can reach up to 60% of power consumption by applying decoupling techniques between air conditioning production and demand [7]. Maximum efficiency will be achieved when every component is optimized for the foreseen thermal demand, including BHE.

The BHE length needed for a given output power greatly depends on subsurface characteristics, such as temperature, particle size and shape, moisture content, and heat transfer coefficients. Due to these factors the completion of a thermal response test (TRT), determining ground thermal properties, is very important. Correct sizing of the BHE is a cause for design concern. Key points are building load, borehole spacing, borehole fill material and site characterization. Over-sizing carries a much higher penalty than in conventional applications. The importance of having TRT techniques is illustrated by the initiative of the Energy Conservation through Energy Storage (ECES), Implementing Agreement (IA) of International Energy Agency (IEA), to launch in 2006 the Annex 21, Thermal Response Test [8].

The TRT was first developed in Sweden and the USA in 1995 [9,10] and is currently used in many countries for sizing BHEs [11]. The standard TRT consists in injecting a certain heat load inside the BHE and measuring changes in the input and output temperature of the circulating fluid. A lot of effort has been put on accurate calculation of subsurface thermal conductivity and borehole thermal resistance from the TRT data using both, analytical or numerical models [12–16] and laboratory experiments. Moreover, a sensible aspect of the measuring process is to keep constant the heat injection or extraction. Recent studies seem to suggest that, under some circumstances, the uncertainty of the heat injection rate can produce a significant uncertainty related to the analysis of thermal conductivity for a typical TRT [17].

The impact of ambient temperature in TRT measurements has also been studied, and it has been proposed a method to filter this effect in the results of conventional TRT [18]. The standard TRT assumes that the subsurface structure is homogeneous but, in real GHP installations, that is the exception and not the rule. The knowledge of the subsurface geological structure may have some importance for a proper sizing of the BHE length in order to minimize the deployment costs, especially for large GHP systems [19]. For instance, using this information it could be possible to stop the boreholes before they reach a weakly conductive layer, and use more shorter boreholes instead of fewer long boreholes, increasing the system efficiency and reducing the deployment costs, With this idea in mind, several works have explored alternative methods to perform TRT but measuring the temperature distribution along the U-tubes. Hurtig *et al.* [20], Acuña [21,22] and Fujii *et al.* [23,24] measured the temperature along the U-tube inner side by using optical fiber temperature sensors based on Raman scattering. Fujii found a good agreement between the calculated thermal conductivity and the local geological and groundwater conditions. However, the optical equipment required by this technique is sophisticated and expensive, and the procedure for introducing the optical fiber inside BHE is relatively complex and delicate. Moreover, the use of this kind of optical equipment is limited because a trade-off between distance, time and temperature resolutions is required [21].

Rohner *et al.* [25] have developed an electronic sensor probe equipped with temperature and pressure sensors that can be introduced inside the BHE to acquire temperature profiles along one of the two U-tubes. Once recovered, the probe can dump the data to a personal computer. This method is based on a measurement of the geothermal gradient and assumes that the terrestrial heat flow is known at the area where the test is performed. The application of the method is therefore limited because detail heat flow maps with sufficient heat flow measurements are not available in many regions of the world including, for example, parts of the US and Canada. In this work, a novel wireless sensor system for measuring the thermal fluid temperature inside pipes during a TRT is presented. It is based on a small, wireless and low-power autonomous sensor. Once configured, this sensor can be easily introduced inside the BHE and it flows with the thermal fluid along the whole U-tube length. After extraction, the sensor establishes a wireless connection with a laptop computer and downloads all the measured thermal data. The sensor dynamically measures the BHE temperature profile during the heat injection, providing extremely valuable information for TRTs that is currently not been provided by any other technique with such a high spatial, temperature and time resolution. Moreover, only one probe is required to get the temperature profile, and it can be reused several times to perform new measurements even in different GHP facilities.

## 2. System Architecture

The aim of the sensor system is to determine the spatial and temporal behaviour of the fluid temperature along the BHE, so it is necessary to measure the temperature of the fluid flowing through the pipe along the entire length. To do so, miniature temperature probes are introduced inside the BHE tube to measure the temperature at specified intervals. The required system features are: integration capability into standard TRT equipment, easiness of use of the sensor system (configuration, acquisition and data storage) and complete independence between spatial resolution, temporal resolution and sampling time. To meet these requirements it will be used “autonomous wireless smart sensors” technology. This technology simplifies both the autonomous sensor configuration and data downloading steps performed by the operator because they are based on specific software. Moreover, it will also facilitate the mechanical sensor handling because the absence of electric terminals and the sphere watertightness. Besides that, it includes a simple and cost-effective temperature sensor, based on Pt100, that will allow to eliminate the dependence between spatial, temporal and temperature resolution shown by other sensor systems, as those based on fiber optics.

The sensor system includes the autonomous sensor, the software for sensor configuration, storage and processing of the thermal data, and two special valves for insertion and extraction of the autonomous sensor into the BHE pipe. The sensor has to be inserted manually in the U-tube using the special valves each time the user wants to measure a temperature profile.

**Figure 1.**
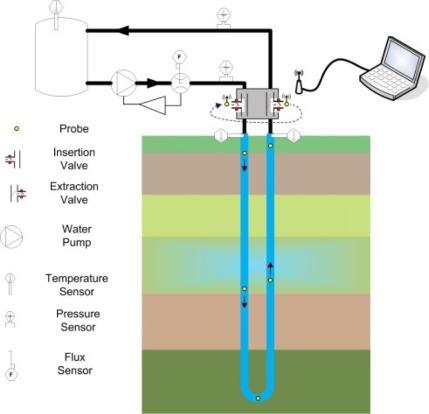
Schematic of BHE and sensor system.

Figure 1 presents a schematic of the sensor system in a BHE installation; the hydraulic system comprises a water tank, a circulation pump and two valves for the insertion and extraction of the autonomous temperature probes. A laptop runs the program for TRT configuration, acquisition and analysis of the temperature data. Finally, a set of small 25 mm diameter balls contains the electronic circuitry of the autonomous temperature probes.

Also, a set of sensors monitor several variables during the running of TRT, such as the flow of water that circulates, the inlet and outlet water temperature of BHE, the temperature of the tank, as well as the pressure in the pipes.

### 2.1. Autonomous Sensor

**Figure 2.**
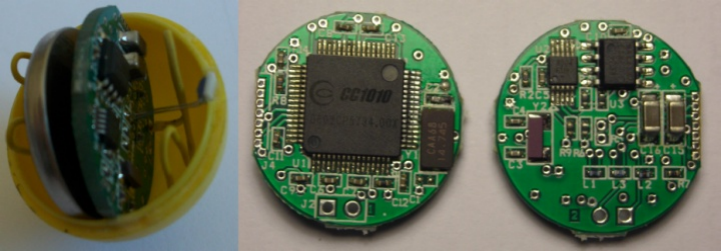
Autonomous sensor inside the POM sphere (left) and detail of the electronic circuitry (right).

The autonomous sensor is the key component of the sensor system. It is an electronic device that measures the thermal evolution of an elementary volume of water along the BHE pipe. Its size must be as small as possible so they can move easily through the pipes carried by the water flow but, at the same time, its size has to be enough for containing an acquisition system, temporary storage and unloading capability of temperature data. To achieve these functions, an electronic circuit has been designed based on the CC1010 transceiver that can be packaged in a polioxymethylene (POM) sphere with diameter smaller than 25 mm. It has been designed a 4-layer PCBs for mounting all the necessary components, (see Figure 2). The characteristics of the autonomous sensor are:

- Temperature range: 0–40 °C
- Resolution temperature: <0.05 °C
- Accuracy temperature: ±0.05 °C
- Sampling interval: 0.1–25 s
- Sampling capacity: 1,000 samples

The mode of operation of the autonomous sensors is as follows: First, the control system selects an available probe and changes its status from low-power to configuration mode. Then, the parameters of sampling are transferred to the probe, and the probe is inserted into the BHE water flow. The probe automatically starts the process of acquiring and storing water temperature at fixed intervals. Once the travel along the whole BHE pipe is completed, the probe is extracted, the temperature data are downloaded to the control system and the probe goes to low-power mode.

In order to accurately determine the position of the sensor inside the pipe, it is needed to ensure that the sensor is carried by the water flow at the same speed. This is achieved by controlling that the density of the sphere that constitutes the probe is very close to the density of the thermal fluid. In most cases the thermal fluid is water, so the spherical probe of 25 mm diameter has been engineered for a total weight of 8.2 gr, thus its density is exactly 1.002 gr/cm^3^. Under these circumstances, knowing the flow rate and the pipe internal diameter (32 mm) is immediate to calculate the speed of the autonomous probe and, consequently, its position. Experimental tests have demonstrated that, under these circumstances, the error in determining the probe position along the tube is less than 2% for all the flow rates considered (700–1,300 L/h). The probe has been tested until a pressure of 4 atmospheres, but its elevated wall thickness/radius ratio (wall thickness is 1.5 mm and radius is 12.5 mm, so ratio is 12%) and its spherical shape suggest that it can operate properly under much more elevated pressures. The key factor for success in using these autonomous probes lies in the duration of the power supply. Each probe carries a battery type button 3 V and 68 mAh, which guarantees the proper functioning of the probes during more than a year for the duty cycle they perform. The management of work and rest cycles and the use of 14.7456 MHz and 32.768 kHz crystals during periods of wireless communication and capture temperature, respectively, are fundamental to achieve the duration theoretically calculated.

Moreover, the electronic circuit in which the temperature sensor is integrated has also been designed to minimize consumption and maximize the miniaturization and precision, using an energy management protocol that keeps the autonomous sensor on low-power state except during the short periods of configuration, acquisition and data download.

**Figure 3.**
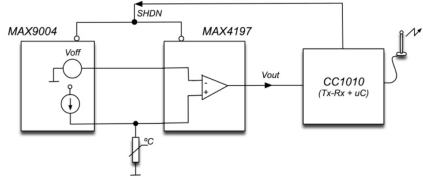
Block diagram of temperature conditioning circuit.

The temperature sensor element is a planar Pt100 from TC Direct®, A-class according to IEC 60751, mounted on a ceramic substrate of size 2 × 2 × 0.4 mm. The polarization current was set to 1 mA, and it is provided by a power source based on a MAX9004 low-power operational amplifier IC. Output signal conditioning is performed by a MAX4197 instrumentation amplifier IC, as illustrated in Figure 3. Both ICs are enabled by a shutdown signal that keeps the electronic circuit in a low-power state when is not acquiring temperature data. The shutdown signal is controlled by the power management system implemented on the sensor microcontroller.

Each sensor is individually calibrated by means of a thermal bath FRIGITERM 600038 from PSELECTA®. A high accuracy thermal probe Pt100 from TC Direct®, class 1/10 according to IEC 60751, is used as reference. The use of an AGILENT U3402A high precision digital multimeter provides the required accuracy for proper calibration.

The microcontroller inside the transceiver is responsible for management of communications, data acquisition and power management. The firmware developed has four states: “power down”, “configuration”, “in acquisition” and “download”.

The autonomous sensor is in “power down” state while waiting to be used for a measurement. A reset signal wakes-up the microcontroller and sets its state to “configuration”. In this state, a wireless communication session is established between the autonomous sensor and the PC to identify the sensor and to configure it to make the measurements. The protocol is very simple in order to achieve high energy efficiency and power savings. The configuration messages are short and the acknowledgments are minimized. This is possible because distances between transceivers are very small, so error rate is low.

When the configuration is completed, the sensor changes to “in acquisition” state. Along the pipe, the autonomous sensor remains in sleep mode and changes to “acquisition” mode only when is required to perform a measurement, according with the previously loaded configuration. When acquiring, the sensor makes a measure, saves the temperature in internal memory, and puts the microcontroller in sleep mode again.

When all measurements are completed, the sensor changes to “download” state. In this mode the sensor establishes a wireless connection with the laptop and transfers all the stored data. Once the transfer is completed, the autonomous sensor is put in “power down” state again.

Due the fact that the sensor is enclosed in a waterproof POM case, is impossible to change the power source. Experimental test has been conducted in order to verify the number of measures that the autonomous sensor can perform. Using a coin Lithium battery CR1620 as shown in Figure 2, a total of 189 measures cycles have been completed, each cycle consisting of 24 temperature measurements.

### 2.2. Software

**Figure 4.**
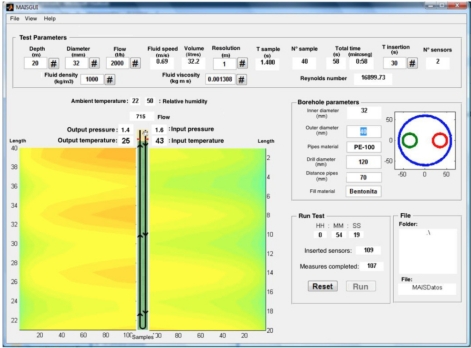
GUI for the configuration, storage and analysis of the TRT data.

It has been developed a program for PC that controls the configuration, execution, data storage and graphical plotting. The graphical user interface (GUI) is showed in Figure 4. It has been developed using Matlab®.

The program asks for information about the physical parameters of the BHE, as borehole depth and diameter, fluid density and viscosity, flow rate, *etc*. And according to the desired number of sampling points, the program establishes a wireless connection with the autonomous sensor unit to load the proper configuration. Once the measurement is completed, a new wireless connection is established and the thermal data is downloaded and stored. Finally, the program process the stored data to obtain information used for BHE characterization.

## 3. Laboratory Tests

**Figure 5.**
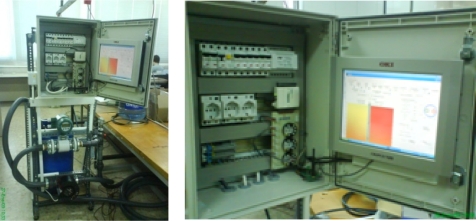
BHE subsystem prototype.

**Figure 6.**
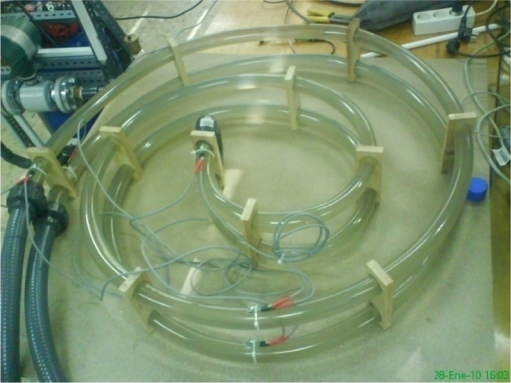
Laboratory 5 m U-tube spiral layout.

Intensive laboratory tests were performed to ensure the proper behavior of the autonomous unit and the required software. A laboratory installation was built to simulate a BHE, as shown in Figure 5 and Figure 6. The hydraulic circuit comprises a water tank, as buffer for the thermal fluid, an electronically controlled circulation pump, a flow meter and two valves, one for inserting probes and another for their extraction. The water temperature can be set through an electric heater controlled by the program that runs on the PC, which also controls the flow of water that is injected into the pipe of BHE. When extracted, the probe is situated at the point of data discharge and, once it is completed, the data contained in the probe is deleted and, the probe is ready for next insertion.

A 5 m long U-pipe has been arranged in spiral layout to perform several tests regarding transit time verification, response time of the temperature sensor and correlation of temperature measurements between the probes and the thermal fluid.

Using this setup, it has been experimentally validated that the autonomous sensor speed inside the U-tube is the same that the speed of the water flow, obtaining a position error smaller than 2% for all the flow rates considered (700–1,300 L/h).

Additionally, since the autonomous probe is continuously moving along the tube while performing the temperature measurements, the response time of the element used as temperature sensor (Pt100) must be precisely known to correlate with the values of temperature measuring point. According to the experimental measurements, the response time is quite small, being 0.5 s to reach 66% of the final temperature, and 1.5 s to reach 90%. The autonomous sensor speed depends on the flow rate and the pipe section and, for the flow rates considered, its value is between 24 cm/s (flow rate of 700 L/h) and 45 cm/s (flow rate of 1,300 L/h). This means that during the time required for the temperature sensor to stabilize, the autonomous probe has travelled 70 cm (worst case, 45 cm/s × 1.5 s). Given than, typically, the temperature change along 70 cm inside a borehole is very small, it can be considered that this effect is negligible and temperature measurement error is only dependent on the sensor resolution.

## 4. Field Tests

**Figure 7.**
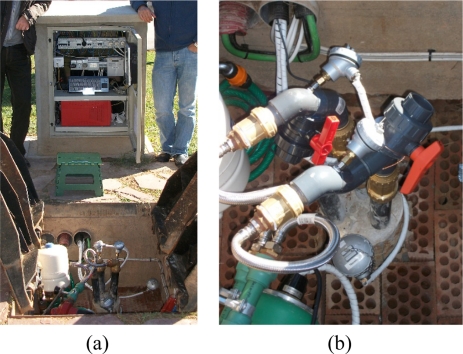
Image of the field test installation (**a**) and detail of the insertion/extraction valves (**b**).

A 30 m deep borehole with a polyethylene (PE100) U-pipe of 30 m length has been drilled on the main campus of the Universidad Politécnica de Valencia. Both pipes are kept 100 mm far away by installing polyethylene separators every 2 m deep. The BHE length is modest because of budget constraints, but it is deep enough to test the sensor system and to evaluate novel methods and analysis techniques. The diameter of borehole is 160 mm and the geological profile presents five layers with the piezometric surface at 3 m depth. The well was filled with grout after U-pipes insertion. A 3 kW electric heater and a circulation pump, allow us completed injection test of heat, while input and output temperatures (accuracy of 0.1 °C) and water flow (accuracy of 1%) are recorded. Figure 7(a) presents an image of the field test installation.

By means of special valves, the autonomous sensor can be inserted (and extracted) into (from) the BHE pipes while the water is flowing during a TRT. Figure 7(b) shows an image of these special valves in the BHE installation. It is needed to remark that these special valves are the only difference between the BHE used in this study and a “real” BHE. This fact is very important because it means that this technique can be applied to any BHE installation just by adding the insertion/extraction valves, an easy modification that can be done at a much reduced cost. These special valves are just commercial PVC ball valves modified, and they do not change at all the flow rate. The modification consists on the lateral attachment to the BHE pipe and the blockage of one side of the internal valve chamber. The modified valves are reusable in different BHE installations, so they have a small impact on the total instrument cost.

**Figure 8.**
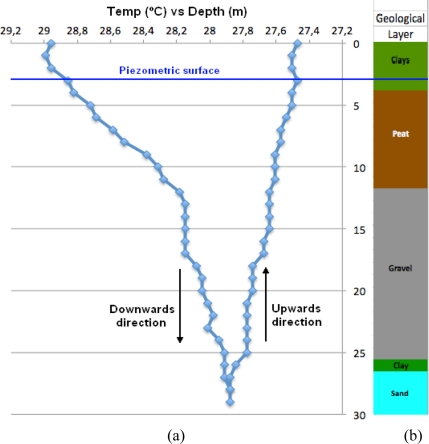
Thermal data as a function of borehole deep (**a**) and comparison with geological subsurface structure (**b**).

On January 20, 2011, during a heat injection test of 2 kW and 2 h after the start, the autonomous sensor was inserted into the U-tube of 30 m. The sensor was configured to acquire the temperature every meter and the flow was set to 725 L/h. Figure 8 shows the data acquired by the autonomous sensor along the pipe. The geological subsurface structure is also illustrated.

As can be seen from the Figure 8, this technology provides information that cannot be obtained with such a high spatial, temperature and time resolution by any other technique. It is observed that most of the heat transfer is produced in the first section of the BHE tube (0–12 m), where the subsurface is composed of clays and peat. In the following section (12–25 m) the heat transfer is much reduced, coinciding with a zone of gravels. Finally, the heat transfer in the last section (>25 m) is almost null. Although the experimental data seems to suggest some correlation between the geological materials and heat transfer, the existence of this correlation cannot be confirmed because the thermal conductivity of each layer is not known. Additional work has to be performed in order to validate this correlation. However, it has to be remarked that the objective of this research work is not to perform a complete geothermal study of this BHE facility but to demonstrate the proper operation of the developed instrument and to show its capabilities regarding TRTs. It can also be observed that the heat transfer in the downwards direction is greater than in the upwards direction, according to other experiments as those performed by Acuña *et al.* [22], because the thermal difference between the heat fluid and the ground is smaller, and, possibly, because it can be some direct heat transfer between both U-tubes [22]. So, from this experiment it can be concluded that, regarding the heat transfer, the most efficient section is the first (from 0 to 12 m). It can also be concluded that the heat transfer efficiency from 25 m downwards is extremely small. This heat transfer analysis is of great interest for the right sizing of the BHE installations. For instance, in this case the autonomous sensor measurements show that it is very inefficient to install BHE longer than 25 m. This kind of information can produce an important increase in system efficiency and, as a consequence, a significant reduction of deployment costs.

## 5. Conclusions

The design of more efficient GSHP systems tailored to subsurface conditions requires new tools and methods for calculating thermal subsurface properties, especially in the case of large BHE systems [19]. For the expansion of these systems it is essential to develop simpler and more economic methods in both, time and money, regarding BHE sizing. The sensor system presented in this work contributes to this issue by offering the possibility of measuring BHE thermal properties inside the U-tubes dynamically, while performing the TRT. Moreover, the sensor system is easily portable and installed, and small in size.

It has been verified in both laboratory and field tests, that it is possible to insert and extract the small probes, containing a miniaturized acquisition system, for temperature monitoring of the water flowing along the pipes of the BHE. The probes are properly configured by wireless transmission. The probe completes the acquisition along the whole U-tube and, also by wireless transmission, downloads the acquired data to a PC. The data collected and recorded provides information about heat exchange efficiency as a function of borehole depth. This information is extremely valuable for the right sizing of BHE. It allows the study and quantification of subsurface effects usually hidden for conventional TRTs, such as underground water flows, the effects of convective layers, *etc*. Additionally, this sensor system offers a spatial resolution lower than 1 m, allowing the optimization of BHE installations in geological areas where the geologic subsurface structure varies with depth significantly. This cannot be done using other techniques that offer a worse spatial resolution [22].

Finally, the data provided by the wireless sensor system opens the door to detailed quantitative assessment of the subsurface thermal conductivity as function of depth. Additional work needs to be done in the future because this method has still a large potential for further development. This additional work concerns the development of new TRT models and analysis tools adapted to the thermal data provided by this novel instrument.
